# Reaching the steady state: 30 years of *Anguillicola crassus* infection of European eel, *Anguilla anguilla* L., in Northern Germany

**DOI:** 10.1017/S0031182024000039

**Published:** 2024-03

**Authors:** Patrick Unger, Johanna Schmidt, Malte Dorow, Sören Möller, Harry Wilhelm Palm

**Affiliations:** 1 Aquaculture and Sea-Ranching, Faculty of Agricultural and Environmental Sciences, University of Rostock, Justus-von-Liebig-Weg 6, 18059 Rostock, Germany; 2 General and Specific Zoology, Institute of Biological Sciences, University of Rostock, Universitätsplatz 2, 18055 Rostock, Germany; 3 Faculty of Biology and Psychology, Georg-August-University Göttingen, Wilhelmsplatz 1, 37073 Göttingen, Germany; 4 Mecklenburg-Vorpommern Research Centre for Agriculture and Fisheries (LFA-MV), Fischerweg 408, Rostock 18069, Germany

**Keywords:** endangered species, European eel, long-term data, parasite–host interaction, swim bladder nematode

## Abstract

A 30 years long data series on the infection dynamics of European eel (*Anguilla anguilla* L.) with the non-native invasive nematode *Anguillicola crassus* Kuwahara, Niimi & Hagaki, 1974 is presented. Parasite burden was evaluated for 30 years in inland and coastal waters in Mecklenburg-Western Pomerania from 1991 to 2020. The total prevalence, mean intensity and damage status of the swim bladders were very high during the first decade (1991–2000), and significantly decreased in both marine and freshwater eel populations in the following decades (2001–2010, 2011–2020). The parasite intensity of eels in coastal waters was significantly lower compared with the freshwater systems (61.3% *vs* 79.5% in the first decade), indicating the vulnerability of the parasites to brackish water conditions and the fact that the life cycle of *A. crassus* cannot be completed under high saline conditions. Eel caught in the western part of the Baltic Sea (west of Darss sill) had the lowest mean infection (51.8% in first decade) compared to the eastern part with 63.8%. Thus, besides different infection patterns caused by the environmental conditions, a temporal trend towards a reduced parasite intensity and a more balanced parasite–host relationship developed in the 30 years of interaction after the first invasion. Possible reasons and mechanisms for the observed trends in parasite–host interactions are discussed.

## Graphical abstract

**Figure:**
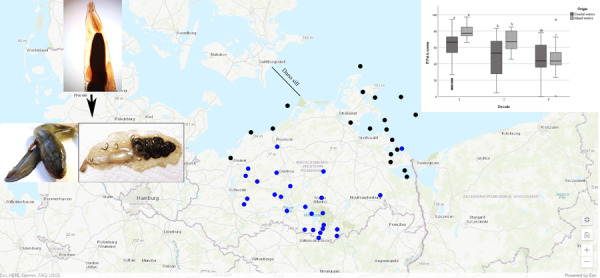

## Introduction

The European eel, *Anguilla anguilla* Linnaeus 1758, is one of the most important fish species for European fisheries and aquaculture. Traditional eel aquaculture in Europe started in the Mediterranean, notably in Italy, in the form of extensive pond cultivation (Heinsbroek, 1991). First aquaculture production in Germany was established in the late 1980s in heated effluents and recirculation aquaculture systems (Heinsbroek, 1991). However, all kind of aquaculture activities depended on wild caught and imported glass eels from France, Italy, UK, Portugal or Spain (Gousset, 1990; Dekker, 2003). Therefore, in 1980, first glass eels of the Japanese eel, *A. japonica* Temminck & Schlegel, 1846 were imported into European aquaculture systems (Kirk, 2003).

In contrast to eel culture, eel fishing has a very long tradition all over Europe. First documented management and stocking activities in Germany date back to the late 1800s century, while landing numbers have been recorded since the early 1900s century (Dekker, 2019), with highest landings in the 1950th. Commercial eel landings in Europe dropped significantly from their peak in the late 1970s to the early 2000s (ICES, 2021). Referring to ICES North Sea recruitment index (ICES, 2021), the numbers of arriving glass eels are currently below 2% compared to the recruitment level before 1980. In a similar magnitude the recruitment dropped in other parts of Europe, where the current level equals less than 10% of the recruitment strength before 1980 (ICES, 2021). The European eel is considered critically endangered by the IUCN in 2008 (International Union for Conservation of Nature and Natural Resources) (Jacoby and Gollock, 2014). To counteract the drastic decline and critical stock situation, a broad discussion on possible reasons and preventive measures were initiated. For example, to cumulate the European eel conservation efforts, the European Union implemented an eel recovery plan in 2007 with new measures for stock recovery (Council regulation (EC) no. 1100/2007).

*Anguillicola crassus*, Niimi & Hagaki, 1974 is a common parasite of the Japanese eel, reaching regular prevalences of infection between 24.5–40.0% along the Japanese coast (Nagasawa *et al*., 1994). According to Koops and Hartmann (1989), *A. crassus* was introduced to Europe in the early 1980s, most probably from Taiwan through stocking for the aquaculture industry. It was then reported for the first time in Northern Germany in 1982 (Neumann, 1985; Spangenberg and Reinhold, 1992), and rapidly became the predominant parasite of *A. anguilla* over most of Europe within 10 years (Moravec *et al*., 1993; Kirk, 2003), reaching a prevalence of infection up to 97% in the Havel river in 1986 (Koops and Hartmann, 1989). The eel becomes infected with *A. crassus* by ingesting the intermediate or paratenic hosts, which are copepods and ostracods and diverse fish species, molluscs, amphibians and insect larvae respectively (Emde *et al*., 2014).

Besides overfishing and environmental issues such as pollution, climatic and oceanic changes and barriers preventing up-stream migration in rivers (Kirk, 2003; Friedland *et al*., 2007), the occurrence of *A. crassus* is considered a further possible reason for the observed population decline. This parasite is assumed to have negative impact on the European eel population (Kirk, 2003) by causing the Anguillicolosis, impairing the swim bladder function and thus, inhibiting a successful spawning migration of mature silver eels (Palstra *et al*., 2007; Barry *et al*., 2014). During its spawning migration, the eel must overcome a distance of more than 5,000 km from Europe to the Sargasso Sea. A daily vertical migration from more shallow swimming depth at night, averaging 282 ± 138 m, to more deeper swimming depths during daytime averaging 564 ± 125 m, was observed (Aarestrup *et al*., 2009). This strenuous migration is potentially made impossible by a severely damaged, non-functioning swim bladder (e.g. Barry *et al*., 2014; Dezfuli *et al*., 2021; Simon *et al*., 2023).

We present a long-term data set of the European eel population in the German Baltic Sea and various inland habitats of Mecklenburg-Western Pomerania (MWP), together with statistical analyses of *A. crassus* infection patterns since 1991. European eels were sampled for 30 years to detect the long-term changes. Possible reasons for the observed trends of both, the host and the parasite population developments, are discussed.

## Materials and Methods

### Sample collection and examination

The study is based on various eel related surveys in the north-eastern German state MWP. According to LALLF (https://www.lallf.de/fischerei/statistik/fangstatistik-kuestengewaesser/), annual commercial eel landings in MWP reached a mean of 41.6 t between 2011 and 2020 in coastal waters. Within the same period, the average annual commercial landings in inland waters of MWP were 48.3 t. In inland waters, eel stocking activities were conducted since more than 60 years (Dorow and Paetsch, 2017). In the first place, stocking should ensure the commercial eel fishing. Since the implementation of the European eel regulation, annual stocking is also a management measure to increase the silver eel escapement.

**Figure 1.**
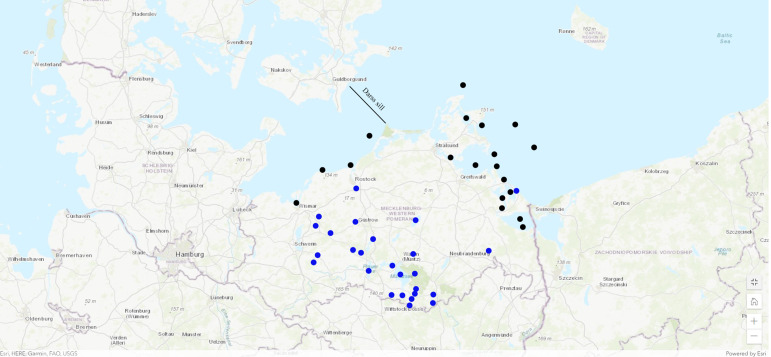
Map with marked sampling points of European eel in Mecklenburg-Western Pomerania. Blue dots show freshwater samples, black dots are sampling locat from the Baltic Sea. Darss sill is dividing the western and the eastern part of the German Baltic Sea. (Map by: ArcGis Online, ESRI)

European eels integrated in this study originated from various scientific studies and samplings, such as eel monitoring programmes and commercial fisheries. Data were collected through the scientific staff of the Thuenen Institute of Baltic Sea Fisheries, Rostock (1991–2003) and the Mecklenburg-Vorpommern Research Centre for Agriculture and Fisheries, Rostock. Analysed data were discriminated by the origin of the caught eels and distinguished between inland habitats and coastal water samplings (Baltic Sea group). In inland waters, samples covered 32 different water bodies and streams. Baltic Sea eel were collected from more than 23 sampling locations, 6 of them from the western part of the German Baltic Sea (West of Darss sill) and 17 from the eastern part (East of the Darss sill, see Fig. 1). Details are given in Supplementary Table S1.

Beside developmental stages (yellow eel, silver eel), the mean weight and length were recorded. The swim bladder damage status was assessed by defining 5 degrees of damage according to Hartmann (1994). Value 1 represents a normal swim bladder with no pathological sign, while 5 describes severe damaged swim bladders, with thickened wall and disfunction. Based on the rating of the individual samples, the mean overall damage degree per sampling was calculated as a rough measure for swim bladder damage for each water body:
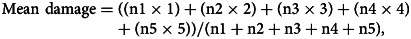
where n1 is the number of eels with damage degree 1, n2 the number of eels with damage degree 2 etc. All eels were included (infected and uninfected).

### Parasitological parameters

The swim bladder of 16 508 specimens of European eel were examined for *A. crassus*. Number of found specimens was counted and the prevalence (%), mean intensity (mI), intensity (I) and mean abundance (mA) were calculated. Parasitological terms follow Bush *et al*. (1997).

### Statistical analyses

Statistical analyses were conducted using IBM, SPSS V.25 (Chicago, Illinois, USA) and were calculated based on the mean values and standard deviation of all eel samplings (*n* = 217). Sampling data were classified into 3 group sets of data, representing 3 decades (D) (D1 = 1991–2000; D2 = 2001–2010, D3 = 2011–2020). Initially, datasets of D1 – D3 were analysed for normality and homoscedasticity and accordingly, the following analyses were adjusted. One-way ANOVA was run in case of normally distributed data to compare the 3 decades in each habitat. In the case of not-normally distributed data, WELCH ANOVA was used. To compare the data by decades, the one-way analysis was followed by post-hoc test, while Tukey's HSD was used in the case of data and Dunnett T3 in case of heteroscedasticity. T-test was run to compare 2 groups, the coastal and inland fish in the same decades. Significance level was set at *P* = 0.05 in all cases. Significant differences between studied means are indicated by letters a, b or c, while the same letters stand for no significant differences between the 2 compared groups.

## Results

### Sampling set up

In total, 16 508 European eel from 217 samplings were investigated between the years 1991 and 2020 (see Table 1). Of these, 12 744 European eels from 147 samplings were caught along the coastline of MWP (Fig. 1). Each sampling included 86.7 fishes in average, with a total length of 48.2 cm in average and a mean weight of 270.8 g. In lakes and streams of MWP, 3,764 European eels were caught from 70 samplings with an average of 53.7 specimens per sample, each. Mean total length was 55.0 cm and mean weight 354.5 g.

**Table 1.** Number of samplings and examined European eel during 3 decades

	Inland waters		Coastal waters	
Decades	No. of samplings	No. of fish	No. of samplings	No. of fish
1991–2000	22	2190	90	9670
2001–2010	14	542	44	2449
2011–2020	34	1032	13	625
Total	70	3764	147	12 744

### Parasitological infection rates

**Figure 2.**
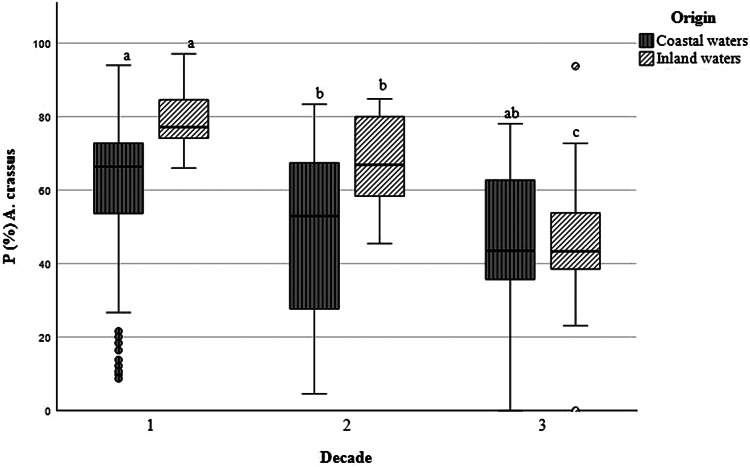
Boxplot diagram of the mean prevalence (*P* (%)) of European eel infected with *Anguillicola crassus* from the Baltic Sea and coastal brackish waters and from inland freshwater habitats during 3 decades. Significant differences between mean prevalences are marked with a, b or c; while 2 groups with same letters do not vary significantly.

During the entire examination period of 30 years, the mean prevalence of eel infected with *A. crassus* was 55.4% (±22.3 s.d.) in coastal and 60.8% (±20.1 s.d.) in inland waters. The highest average infection rates were recorded during the first sampled decade between 1991 and 2000 in both habitats. Inland caught eels were infected with a mean prevalence of 79.5% (±8.4 s.d.) and eels from the Baltic Sea coastal areas with 61.3% (±19.1 s.d.) (see Table 2). During the second decade, the observed prevalence decreased in coastal waters to 46.2% (±24.5 s.d.) and in inland waters to an average of 66.8% (±13.1 s.d.). During the last decade between 2011 and 2020, the prevalence in coastal waters remained at a mean value of 45.6% (±21.8 s.d.) while the prevalence of infection in eels from inland waters further decreased to 46.1% (±16.2 s.d.). For inland waters, the observed changes in prevalence of infection were significant between all three decades. In coastal waters, the prevalence of infection between decades 1 to 2 was significantly different (*P* < 0.05). Between D1 and D3 and D2 and D3 no significant differences were observed (Fig. 2). Overall mean prevalence of all studied eel samples from both habitats continuously decreased over time from 64.9% (D1) to 51.2% (D2) and 46.0% (D3).

**Table 2.** Results of Prevalence (*P*%), mean intensity (mI), intensity (I), mean abundance (mA) and mean Hartmann-class (mHC) (±s.d.) of infection with *Anguillicola crassus* in the swim bladder of European eel for inland and coastal areas in Mecklenburg-Western Pomerania given over time

Time habitat	1991–2000				2001–2010				2011–2020			
	*P* (%)	mI (I)	mA	mHK	*P* (%)	mI (I)	mA	mHK	*P* (%)	mI (I)	mA	mHK
Inland waters	79.5 ± 8.4	8.8 ± 4.3 (4.0–22.4)	7.2 ± 4.1	2.6 ± 0.5	66.8 ± 13.1	5.7 ± 1.9 (3.3–8.3)	3.7 ± 1.3	1.7 ± 0.3	46.1 ± 16.2	3.8 ± 1.7 (1.0–9.8)	1.9 ± 1.5	1.4 ± 0.4
Coastal waters	61.3 ± 19.1	6.3 ± 2.8 (1.4–14.4)	4.0 ± 2.6	2.1 ± 0.6	46.2 ± 24.5	5.8 ± 2.9 (1.0–12.3)	2.9 ± 2.4	1.4 ± 0.3	45.6 ± 21.8	5.6 ± 2.8 (3.5–11.2)	2.9 ± 2.1	1.5 ± 0.4

**Figure 3.**
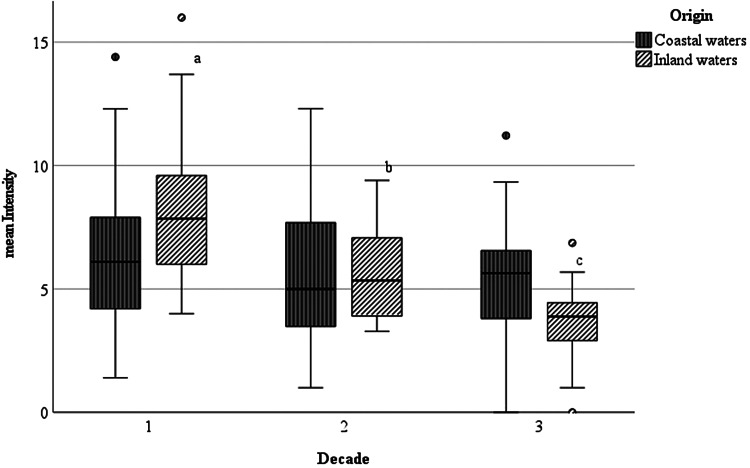
Boxplot diagram of mean intensity (mI) of European eel infected with *Anguillicola crassus* from the Baltic Sea and coastal brackish waters and from inland freshwater habitats during 3 decades. Significant differences between the mean intensity are marked with a, b or c; while 2 groups with same letters do not vary significantly.

The mean number of nematodes per infected eel ( = mean intensity) was highest during the first decade. Altogether, 62 852 specimens of *A. crassus* were found, with 1 to 220 parasites per infected eel. Similar to the prevalence of infection, inland caught European eel showed the highest number of *A. crassus* per individual, with 8.8 (±4.3 s.d.) parasites compared with 6.3 (±2.8 s.d.) in coastal waters during D1. The values decreased during the studied periods of 3 decades and the lowest intensity was observed during D3 between 2011 and 2020 in inland waters, where only 3.8 (±1.7 s.d.) nematodes per infected eel were found. The observed decrease in intensity was highly significant for inland waters during all three decades (*P* ⩽ 0.001) (Fig. 3). In coastal waters, the intensity only slightly decreased to 5.6 ± 2.8 in D3. No significant differences were found here.

**Figure 4.**
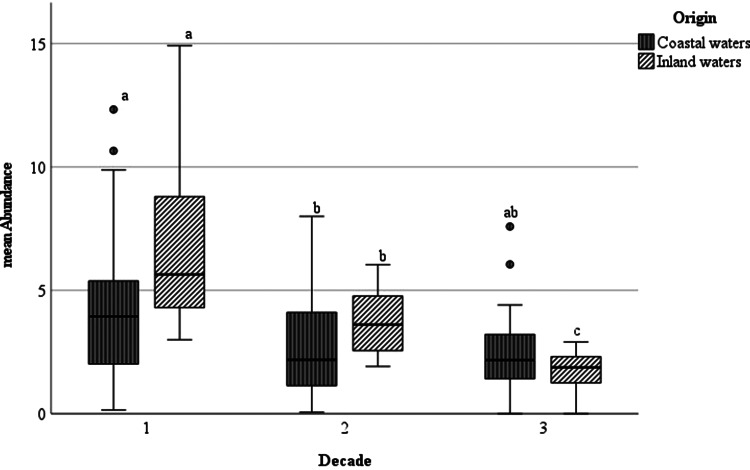
Boxplot diagram of mean abundance (mA) of European eel infected with *Anguillicola crassus* from the Baltic Sea and coastal brackish waters and from inland freshwater habitats during 3 decades. Significant differences between the mean abundance are marked with a, b or c; while compared values with same letters do not vary significantly.

By comparing the mean number of *A. crassus* for all sampled eel (mean abundance) including non-infected specimens, the observed trend of distribution of the mean intensity and prevalence could be confirmed. The highest mean abundance was found during the first decade (1991–2000) in the inland waters (7.2 *vs* 4.0), while between 2011 and 2020 the lowest mean abundance was observed (1.9) (Table 2). For eel from inland waters, all differences between the decades were significant, while those from the Baltic Sea only showed significant differences between D1 and D2 (*P* < 0.05) (Fig. 4).

**Figure 5.**
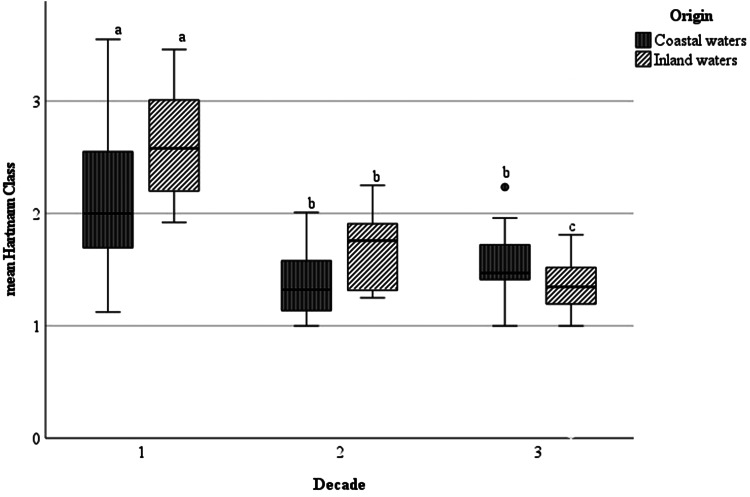
Boxplot diagram of mean Hartmann-class (mHC) of European eel infected with *Anguillicola crassus* from the Baltic Sea and coastal brackish waters and from inland freshwater habitats during 3 decades. Significant differences between the mHC are marked with a, b or c; while compared values with same letters do not vary significantly.

The mean Hartmann-class (mHC) followed the same trend as the other parasitological parameters. All values continuously decreased over time, with significant differences between all 3 decades in inland waters (Table 2). The highest average damage status was found during the first decade in eels caught in freshwater (2.6 ± 0.5) and brackish water (2.1 ± 0.6), respectively. Within the following decades, a strong and significant decrease of the mean damage status was especially observed in freshwater. For damage status of eel from the coastal waters of the Baltic Sea, only for D2 and D3 no significant differences were observed (*P* = 0.686) (Fig. 5), where the mean Hartmann-class was relatively low during both decades (1.4 and 1.5).

## Discussion

The present study analysed a 30 years long data series of the infection rates, damage and thus parasite–host relationship of the neozoon parasite *A. crassus* (Nematoda) in its new host European eel in German (MWP) coastal and freshwater habitats. During the first decade, starting appr. 10 years after the first occurrence of this parasite in German waters, the parasite prevalence, mean intensity and abundance reached highest values. Because *A. crassus* was introduced to Germany in the 1980s, the first years after the introduction event to Europe represented a period of very high infection rates. The European eel, a naive definitive host, was very susceptible to the infection with the invading nematode *A. crassus* and seemed to have no or little defence mechanisms (Kirk, 2003). Studies by Koops and Hartmann (1989), Möller *et al*. (1991) or Sprengel and Lüchtenberg (1991) indicated that the first infections with *A. crassus* in water bodies in Northern Germany occurred between 1985 and 1987, reaching a prevalence of 4% (in 1986, North Sea, near Helgoland) – 97% (in 1986, Havel River). The best studied region during this period was the lower Elbe river, where no infection was found in 1985, while 1 year later the rate already varied between 6 and 27% (Koops and Hartmann, 1989). Between October 1987 and September 1988, the average infection with *A. crassus* reached 57.7% (Möller *et al*., 1991), which is similar to our observed average prevalence rate of 61.3% in coastal waters within the first decade (1991 to 2000).

The mean overall prevalence of *A. crassus* in the eel during the first decade was 79.5% in inland waters. This demonstrates a generally higher infection rate in freshwater ecosystems compared with brackish water ones. Although the number of samples taken in inland and coastal waters is not the same, the statistical comparison is still significant because the number of eels in each decade is at least 500. In an earlier long-term study on *A. crassus* infections in European eel from Northern Germany between 1996 and 2011 by Wysujack *et al*. (2014) a continuously high prevalence of approximately 70–85% in several inland waters in Northern Germany (river and lakes) and in the Baltic Sea were reported. The authors observed no trend of decreasing infection rates over time. Studies in Belgium (river in Flanders) and France (in oligosaline Fumemorte Canal in Camargue) described the settlement of *A. crassus* as a 2-stage pattern, consisting of a rapid spread with increasing prevalences during the first years followed by ceiling levels at 60–70% (Audenaert *et al*., 2003; Levebvre and Crivelli, 2004). Similarly, a stabilization of the *A. crassus* prevalence at levels of ~60% was reported by Schabuss *et al*. (2005) in the Neusiedler See (Austria) and in Lake Balaton (Hungary). A similar decreasing trend is herewith reported from inland waters during the last decade of the years 2011 to 2020. Compared to the second decade, a harsh reduction of the infection rate by more than 20% was observed. In coastal waters, the prevalence decreased slightly and stabilized at an average level of 45.6% (see Table 1). In total, a massive reduction of prevalence by around 33% in inland waters during this 30-years of long-term analysis was observed. In coastal waters, the reduction was less clear with ca. 16% over 30 years. Interestingly, all these prevalence of infection data seem to not correspond to another long-term data set from Portugal. Pereira *et al*. (2022) studied European eel from Minho River (Portugal) over a period of 26 years between 1995 and 2021. In general, the mean prevalence was lowest during the first decade (30.7%, 1995/96), increasing to 67.9% during the second decade (2009/11), subsequently followed by a slightly decrease to 63.1% during the third decade (2017/21). Because the authors stated that the first infection of *A. crassus* in Minho River was recorded in 1995, their study covered the immediate beginning and development of the infection. Thus, in case of the Minho River, the infection of *A. crassus* in the European eel started with an initial phase at low prevalences and remained on a similar high infection level as observed from other regions (see above) during the following years from 1995 to 2018. These results seem to contrast our data, concerning the overall peak of infection and the long-term development. However, as stated by Koops and Hartmann (1989), also the infection in German waters needed some years to reach its peak, similar to the first period identified by Pereira *et al*. (2022). On the other hand, the peak reached in Germany was much higher (up to 100%) compared with Minho River (67.9%). A possible reason for this difference might be seen the different population densities of the host in Minho River. A lower population size at the beginning of the parasite invasion might result in a lower prevalence of infection with *A. crassus* and subsequently followed by a lower adaptation pressure onto the host.

Distinct differences between the infection levels of the Baltic Sea and the inland freshwater environments were observed. The lower prevalence and minor infection intensity in the first and second decade resulted in an overall decreasing damage status of the swim bladders in the coastal waters of the study region. The infection in the coastal waters was clearly lower than in freshwater and thereby the results of former studies were confirmed (see Kennedy, 2007; Székely *et al*., 2009; Wysujack *et al*., 2014). Simon *et al*. (2023) found the same trend in their study, indicating that eel from the open coastal waters have a higher fitness compared to species originating from nearby brackish lagoons. It has been shown that hatching rate, survival and infectivity of the second larval stage of *A. crassus* declines with increasing salinity (Kirk *et al*., 2000). Even though adult parasites can survive in seawater for a certain time and may even produce eggs, the completion of the life cycle in saline waters appears to be limited due to physiological effects of salinity as well as to the fact that many of the marine copepods are of the insuitable size to serve as intermediate hosts (see Kennedy, 2007). Several studies support the absence or rare abundance of *A. crassus* under high saline conditions (Jakob *et al*., 2009; Giari *et al*., 2021).

The mean intensity of infection was also highest during the first decade inside the freshwater habitats with 8.8 ± 4.3 specimens per fish. This mean intensity continuously decreased over time with minimum of 3.8 ± 1.7 in the third decade. The mean intensity in brackish waters was generally lower and showed stabile or only slightly decreasing trends over time. In other German inland waters, early studies like Spangenberg and Reinhold (1992) reported 10–37 nematodes per swim bladder in rivers around Berlin and Potsdam, 5.0–5.3 nematodes per swim bladder in the river Rhine (Sures *et al*., 1999), and 4.2–5.9 *A. crassus* per eel in the river Weser (Reimer, 2000). Similar to our study, the values were highest in the early 1990s and decreased over time. A similar infection history was found in a long-term study at the upper Lake Constance in South Germany. Bernies *et al*. (2011) recorded a drastic initial increase of the infection intensity, which peaked at 16 nematodes per swim bladder 4 years after the first occurrence of the parasite in the lake in 1988. After this peak, the values started to drop rapidly within 2 years. This decrease then slowed down but continued consistently until the end of the study in 2009, when the mean infection intensity was only 3.3 ± 0.5 nematodes per infected eel. The same trend was also observed for the mean abundance.

The observed mHC was highest in the 1990s, indicating moderate impairment of the swim bladders. Within the following years, the damage status significantly decreased in both freshwater and Baltic Sea habitats to lower average values. Wysujack *et al*. (2014) found similar values of the Hartmann classes in Northern Germany, with the highest mean damage degree of 2.71 ± 0.32 in river Weser and the lowest value of 1.05 ± 0.04 in the coastal waters of MWP. Pietrock *et al*. (2002) investigated in total 406 European eel from 6 lakes in Brandenburg in 2001 and found a mHC of 2.6. Other authors used the Swim bladder Denerative Index (SDI) after Lefebvre, Contournet and Crivelli (2002) with values between 0 and 6. Pereira *et al*. (2022) calculated a mean SDI of 2.51 ± 1.12 for eel in Minho River, Portugal.

According to these available data sets and our new data, it seems that the eel population in the third decade was much more adapted to the parasite infection compared with the first and second decade, both with less inflamed swim bladder walls and less infection intensities. In comparison to Pereira *et al*. (2022), it must be considered that our data represent the decades 2–4 after first invasion, where a continuous host–parasite adaptation period following a peak infection level takes place. Several reasons can be argued to explain the observed shift from an infection peak towards decreasing infection levels:

1. *The individual host acquires parasite immunity over time and is able to reduce the number of parasites*. Knopf *et al*. (2008) experimentally infected eel with L3-larvae of *A. crassus* and observed an increased inflammatory reaction in the surroundings of the nematodes in the swim bladder wall. The inflammatory infiltrate consisted of monocytes and macrophages, granulocytes and lymphocytes and was only observed in the surrounding of some of the present *A. crassus* larvae. Thus, an activation of the defence cells resulting in an increased migration activity in infected eels was documented (Knopf *et al*., 2008). Bracamonte *et al*. (2021) stated that the encapsulation of nematodes, which is commonly observed in native hosts, also takes place in the novel host *A. anguilla*. These authors described an increased frequency of encapsulation over time which was positively associated with the reduction in numbers of adult specimens of *A. crassus* inside the eels. The mean infection intensity of adult parasites was 5.1 ± 1.6 in non-capsulating eel compared to 2.0 ± 0.5 in the encapsulating eels.
2. *Heavily infected eels might die after a certain time*. Studies from the early 2000s showed that *A. japonica* has more effective defence mechanisms against *A. crassus* compared with the European eel (Knopf and Mahnke, 2004). Egusa (1979) reported only negligible pathological effects of this parasite onto *A. japonica* but certain mortality among *A. anguilla* cultivated in Japan. In all above cases, a decreasing infection rate must be accompanied by a likewise decreasing infection intensity, as recorded in the present study. The first option was challenged by Denny *et al*. (2013), Neto *et al*. (2010) and Pereira *et al*. (2022), who did not observe any negative effect of *A. crassus* infection on the eel body condition during their continental growth phase. The general body condition seemed to be not negatively influenced by the swim bladder damage caused by the nematode, which was also observed in all other studies, including the present one. Consequently, direct mortality might therefore not explain the observed changing prevalence and infection intensities.
3. *Infected eels die on their way to the Sargasso Sea, only allowing better adapted or lower infected specimens to reproduce, driving towards a better adapted population.* The functionality of the swim bladder still comes into focus when dealing with the long spawning migration of European eel (Palstra *et al*., 2007; Newbold *et al*., 2015) and not during the continental growth phase. Thus, a higher mortality during the spawning migration is feasible caused by observed infection values. As infected eels have to utilize 20% additional energy reserves than they would normally need (Palstra *et al*., 2007), it could prematurely deplete the energy needed to reach the spawning location in the Sargasso Sea. This would lead to the death before mating thus to a reduction of numbers of heavily infected eels taking part at the spawning (Pelster, 2015). Infection with *A. crassus* also impairs the mechanism of gas secretion by severely reducing the level of oxygen in the swim bladder through infection (Kirk, 2003). Metabolic processes that depend on the gas gland cells and thus on gas secretion could also be impaired (Pelster, 2015). In addition, the ability to regulate the eel's capacity to descend to depths in water may be inhibited (Palstra *et al*., 2007). In this sense, it has been hypothesized that full maturation of the reproductive organs does not occur unless individuals swim to dee*per se*a water (Sébert *et al*., 2007; Sjöberg *et al*., 2009). Consequently, parasite infection will result in a loss of potential spawners for the eel population, with individuals dying either on the way to or inside the Sargasso Sea. Therefore, well-adapted eels are likely to be the main contributors to the maintenance of the overall spawning stock.
4. *Parasite avoidance strategies of either the definitive or the intermediate hosts.* Parasites are known to alter the host behaviour in order to have a better transmission to the next host (Palm *et al*., 2018). Consequently, survival of heteroxenous parasites depends on successful strategies to either manipulate the host or prevent detection through the immune system. A neozoic parasite such as *A. crassus* cannot infect its host undetected, without the possibility of adopting avoidance strategies to elude the immune response. This kind of reactions has already been observed by Bracamonte *et al*. (2021) and Knopf *et al*. (2008) and might also play a role resulting in the observed infection patterns. However, most *A. crassus* larvae occur in several species of cyclopoid copepods, which are the obligate first intermediate hosts. Additionally, about 50 paratenic hosts, like several amphibians or insects and at least 37 fish species are known as potential participants of its life cycle (Emde *et al*., 2014). With the very generalistic feeding regime or European eel (Bouchereau *et al*., 2009; Denis *et al*., 2022), such avoidance of intake of hosts seems impossible.

It is interesting to note that, in accordance to the observed decline in parasite intensity in eel during the third decade, studies by Dorow *et al*. (2021, 2023) using different stock assessment methods indicate independently from each other an increased eel population in coastal waters of MWP since 2016. Thus, a higher number of definitive hosts in the region might support increasing infection levels with *A. crassus* in future, placing also more pressure onto the eel defence strategies. This hypothesis could compete with the stabilization of eel stocks through increased host–parasite adaptation. Accordingly, it is recommended to continue the established standardized assessment *A. crassus* to document to parasite infection development in the future. In the face of eel conservation efforts being implemented on a European scale, such data are needed to evaluate the impact of *A. crassus* on individual health status and reproduction potential.

## Conclusions

In summary, our analyses using a 30-year long data set demonstrate that the infection of European eel with *A. crassus* in Mecklenburg-Western Pomeranian inland and coastal waters reached high infection rates during the first 10 years of investigation (D1). This period, however, must be considered already a second phase in the infection dynamics of *A. crassus* in German coastal and inland waters, most probably following a first phase of strongly increasing infection rates until reaching a peak level. Then, the prevalence, mean intensity, mean abundance and swim bladder damage status continuously decreased during subsequent years and in both habitats. Thus, the observed trend suggests that a steady state of infection has been reached in the eel population, with moderate levels of infection.

Of the 4 proposed explanations for the continuous decline in the infection rate, the most plausible one is the increase in the number of individuals with an immune response capacity against *A. crassus*, which was absent in the eel population that suffered a higher mortality during the initial infection period. Because eel from coastal waters tend to be generally less infected with *A. crassus*, we suggest that eel stocks in coastal waters may be of higher value for eel conservation efforts because they seem to be less impaired regarding the spawning migration compared to eels using nearby inland waters as growing habitats. Regarding the number and quality of spawners, the shown differences in the parasite intensity with *A. crassus* within different habitats during the continental life phase of the European eel should be considered for future management strategies.

## Supporting information

Unger et al. supplementary material Unger et al. supplementary material

## Data Availability

The data that supports the findings of this study is available as supplementary materials.

## Supplementary material

The supplementary material for this article can be found at https://doi.org/10.1017/S0031182024000039
